# Pan‐Immune‐Inflammation Value for Mortality Risk Stratification in Critically Ill Patients With Acute Ischemic Stroke: A Cohort Study Based on the MIMIC‐IV Database

**DOI:** 10.1002/cns.70831

**Published:** 2026-03-13

**Authors:** Zhenzhu Li

**Affiliations:** ^1^ Department of Emergency Tianjin Union Medical Center, The First Affiliated Hospital of Nankai University Tianjin China

**Keywords:** acute ischemic stroke, all‐cause mortality, inflammatory markers, MIMIC‐IV, pan‐immune‐inflammation value, risk stratification

## Abstract

**Aims:**

The pan‐immune‐inflammation value (PIV) is an emerging inflammatory biomarker whose prognostic role in critical acute ischemic stroke (AIS) remains unclear. This study aimed to explore the association between PIV and all‐cause mortality (ACM).

**Methods:**

This retrospective cohort study enrolled 710 ICU patients with AIS from the MIMIC‐IV database. The association between PIV and 90‐day (primary), 30‐day, and 1‐year ACM was evaluated using multivariable Cox regression, restricted cubic splines, subgroup analyses, and sensitivity analyses.

**Results:**

Each 1000‐unit increase in PIV was independently associated with an 8% higher risk of 90‐day ACM (hazard ratio [HR] = 1.08; 95% confidence interval [CI] 1.03–1.13; *p* < 0.001). A reverse L‐shaped nonlinear correlation was observed (threshold: 2987.61). The mortality rate was markedly higher in patients above versus below this threshold (52.7% vs. 28.3%). Subgroup analyses further revealed that this association was significantly stronger in patients without sepsis (*p* for interaction = 0.007). Both sensitivity analyses—complete‐case and multiple imputation—yielded consistent results, confirming the robustness of the main findings. The results remained consistent for 30‐day and 1‐year ACM.

**Conclusions:**

PIV exhibited a reverse L‐shaped relationship with short‐ and long‐term ACM in critical AIS, and served as a tool for ACM risk stratification.

## Graphical abstract

This study identified a nonlinear, reverse L‐shaped relationship between the pan‐immune‐inflammation value (PIV) and all‐cause mortality (ACM) in critically ill acute ischemic stroke (AIS) patients, with a distinct threshold for risk stratification. Furthermore, the prognostic value of PIV was significantly stronger in patients without sepsis. These findings establish PIV as a practical and independent biomarker for mortality risk assessment in critically ill patients with AIS.

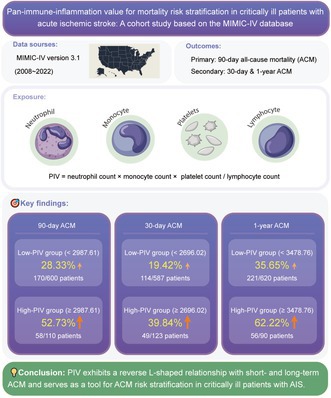

## 1 Introduction

Acute ischemic stroke (AIS) affects millions of individuals worldwide. The Global Burden of Diseases, Injuries, and Risk Factors Study (GBD) 2019 reported that, as of 2019, stroke remained the second leading cause of death and the third leading cause of death and disability combined globally [1]. Stroke imposes a significant economic burden on families, societies, and nations owing to its high incidence, disability, mortality, recurrence, and numerous complications. AIS constituted approximately 2/3 of all incident strokes in 2019 [1]. Therefore, proactive identification of high‐risk patients with AIS, who are likely to experience adverse outcomes, enables clinicians to promptly implement targeted interventions, thereby improving clinical outcomes.

Increasing evidence has indicated a significant association between AIS and inflammation. Neuroinflammatory and systemic immune responses can shape the clinical manifestation and outcomes of stroke [2, 3, 4]. In addition to inflammation in the brain, the detrimental influence of AIS occurs outside the central nervous system, triggering systemic dysfunction in cardiac, endocrine, gastrointestinal, lymphoid, and musculoskeletal tissues [5].

Recently, a systemic inflammatory parameter, termed the pan‐immune‐inflammation value (PIV), has been proposed. It is derived from complete blood count (CBC) tests and is generated by multiplying neutrophil, monocyte, and platelet counts and subsequently dividing the product by lymphocyte levels. The PIV has been extensively used as a biomarker in oncological research. It is a promising predictor of clinical outcome in individuals with advanced cancers, such as colorectal carcinoma, small cell lung cancer, oropharyngeal carcinoma, esophageal neoplasms, and breast carcinoma [6, 7, 8, 9, 10, 11].

The four blood components of PIV correspond to distinct systemic inflammatory responses and immunological signaling mechanisms. PIV, originally validated in metastatic colorectal cancer, outperforms conventional markers such as the neutrophil‐to‐lymphocyte ratio (NLR) and platelet/lymphocyte ratio (PLR) in terms of prognosis [7]. Two other studies found that PIV outperformed the systemic immune‐inflammation index (SII), systemic inflammation response index (SIRI), PLR, and NLR in predicting poor 3‐month outcomes following mechanical thrombectomy for AIS [12] and further outperformed the SII in predicting in‐hospital mortality for AIS patients [13]. Thus, PIV may serve as a more integrated and potent measure of systemic inflammatory burden in stroke patients than other biomarkers.

The predictive power of PIV aligns with the growing consensus in cerebrovascular research that composite and nonlinear biomarker assessments are critical for risk stratification. Recent work underscores the value of multi‐biomarker cumulative burden scores and has revealed complex, non‐linear relationships for single markers like LDL‐C [14, 15]. As a readily available composite index, PIV fits within this advanced assessment framework, capturing a synergistic inflammatory signal that may be more physiologically informative than its individual components.

Given the extensive application of PIV, the significant global health burden of AIS, and their pathophysiological connection to inflammatory processes, this study hypothesized that PIV may serve as a powerful prognostic biomarker in critically ill AIS patients. Specifically, this study aimed to investigate the association between PIV and short‐ and long‐term ACM, with particular focus on characterizing its dose–response pattern (e.g., linear or nonlinear) and its utility for clinical risk stratification.

## 2 Methods

### 2.1 Data Source, Ethical Considerations and Data Privacy

This study employed a retrospective cohort design, using the publicly accessible Medical Information Mart for Intensive Care (MIMIC) IV (version 3.1) database. This dataset comprised de‐identified records of ICU patients treated at the Beth Israel Deaconess Medical Center between 2008 and 2022. This database was approved by the Institutional Review Boards (IRBs) of the Beth Israel Deaconess Medical Center and the Massachusetts Institute of Technology. This study adhered to the Strengthening the Reporting of Observational Studies in Epidemiology (STROBE) guidelines [16]. Moreover, IRB approval was not required for this study as all analyses used a publicly available, de‐identified dataset that had already received prior IRB authorization. Clinical information was retrieved by ZhenZhu Li (Record ID: 65377092), who obtained database access after successfully completing an online certification course.

### 2.2 Study Population

The MIMIC‐IV database comprises the records of 94,458 unique ICU stays between 2008 and 2022. Among these cases, 6791 were classified as having ischemic stroke, as determined by the ICD‐9 and ICD‐10 diagnostic codes (Supplemental Table S1). The exclusion criteria for patients were as follows: (a) non‐first ICU admission; (b) absence of neutrophil, platelet, monocyte, or lymphocyte counts within the initial 24 h of ICU admission, or lymphocyte counts = 0; (c) PIV with abnormal values; (d) length of ICU stay < 24 h; or (e) age < 18 years. Overall, 710 patients met the inclusion criteria and were included in the final analysis (Supplemental Figure S1).

### 2.3 Variable Extraction

All relevant variables were retrieved from the MIMIC‐IV database using the Structured Query Language (SQL) via PostgreSQL (version 17.0) and Navicat Premium (version 16). The data extraction process encompassed six main categories: [1] Demographic variables, including sex, age, and race; [2] Comorbidities, including diabetes mellitus, congestive heart failure, chronic pulmonary disease, sepsis, liver disease, renal disease, and malignant cancer; [3] Vital signs, including heart rate, systolic blood pressure (SBP), diastolic blood pressure (DBP), mean blood pressure (MBP), respiratory rate, temperature, and peripheral capillary oxygen saturation (SpO_2_); [4] Laboratory variables, including white blood cell (WBC) count, hemoglobin, glucose, blood urea nitrogen (BUN), serum creatinine, neutrophils, lymphocytes, monocytes, and platelets; [5] Clinical severity upon admission, assessed using the Simplified Acute Physiology Score II (SAPS II) and Acute Physiology Score III (APS III); [6] Supportive therapies, including mechanical ventilation and vasoactive agent use during the initial 24 h after ICU admission.

The PIV is a composite systemic inflammatory biomarker derived from CBC. This metric integrates the key cellular components of innate and adaptive immunity, calculated using the following formula: “neutrophil count (k/ul) × monocyte count (k/ul) × platelet count (k/ul)/lymphocyte count (k/ul)” [7]. In the present study, the key variable, PIV, was calculated based on the initial CBC test conducted after ICU admission.

### 2.4 Outcomes

The primary outcome of this study was the 90‐day ACM after ICU admission. The secondary outcomes were the 30‐day and 1‐year ACM. ACM was the pre‐specified endpoint; cause‐specific mortality (e.g., cardiovascular vs. non‐cardiovascular) was not available in the MIMIC‐IV database.

### 2.5 Management of Missing Data and Outliers

The missing proportion of the extracted variables was < 5%. All variables with missing values were substituted with the corresponding mean or median values. The PIV exhibits a highly right‐skewed distribution with extremely high values. To ensure the robustness of our findings against any potential outliers, a right‐tail trimming procedure was applied. Specifically, observations with PIV values exceeding the 99th percentile of its distribution (i.e., the top 1% of the PIV values) were excluded from all subsequent analyses. Missing data and outliers were managed using the STATA software (version 18). There were 57 missing values (Supplemental Table 2).

### 2.6 Statistical Analysis

Histogram distribution analysis was performed to evaluate the normality of variables. Because the PIV had an obviously skewed distribution to the left, the values were per 1000‐transformed before analysis. Normally distributed continuous variables were reported as mean ± standard deviation (SD), whereas non‐normally distributed continuous variables were described using median and interquartile range (IQR). Categorical variables were reported as numbers and percentages (%). The chi‐square test was used for categorical variables and the Kruskal‐Wallis H test was used for continuous variables to test for differences among groups.

Mortality was analyzed using Kaplan–Meier (K–M) survival curves stratified by PIV tertiles and compared using the log‐rank test. To examine the relationship between PIV and the primary (90‐day ACM) and secondary outcomes (30‐day and 1‐year ACM), Cox proportional hazards models were applied in both unadjusted and multivariate‐adjusted models. The PIV was entered as a continuous variable (per 1000 units). The results were reported as hazard ratios (HRs) and 95% confidence intervals (CIs). The PIV was converted into a categorical variable based on its tertile distribution, using the lowest tertile as the reference group. Trend analysis was conducted to validate the findings derived from treating PIV as a continuous variable and to explore potential nonlinear associations. The selection of confounders was guided by clinical relevance and prior scientific literature [17, 18, 19, 20]. Multicollinearity among the independent variables was assessed using the variance inflation factor (VIF) method, with a VIF threshold of ≥ 5 indicating the presence of multicollinearity [21]. Three models were constructed for the analysis: Model 1 was non‐adjusted; Model 2 was adjusted for sex, age, and race; and Model 3 was further adjusted for sex, age, race, diabetes mellitus, congestive heart failure, chronic pulmonary disease, sepsis, liver disease, renal disease, malignant cancer, WBC, hemoglobin, glucose, BUN, creatinine, heart rate, SBP, DBP, MBP, respiratory rate, temperature, SpO_2_, SAPS II, APS III, vasoactive agent use, and mechanical ventilation.

Restricted cubic spline (RCS) analysis was employed to investigate any potential non‐linear associations between PIV and survival endpoints. In these models, the PIV was treated as a continuous variable with four knots (5th, 35th, 65th, and 95th knots) as recommended by Harrell. Nonlinearity was assessed using a likelihood ratio test, comparing the model with only a linear term against the model with linear and cubic spline terms. Based on the observed smoothing curve, a two‐piecewise linear regression model was constructed to identify the potential threshold effect with adjustment for potential confounders. Subgroup and interaction analyses were further performed to investigate the relationship of PIV with primary and secondary outcomes, respectively, in relation to several subgroups, including sex, age (< 65 and ≥ 65 years), chronic pulmonary disease, diabetes mellitus, sepsis, vasoactive agents use, and SAPS II (< 40 and ≥ 40). PIV was expressed as a continuous variable (per 1000 units), and if the variable involved in the subgroup analysis was categorical, it was omitted from the analysis.

Sensitivity analyses were performed to assess the robustness of the results. First, individuals with missing values were excluded and the main analyses were repeated in this complete‐case cohort. Second, multiple imputation was applied using the R mice procedure (five imputations with chained equations) and the main analyses were rerun on the imputed datasets.

The predictive performance of PIV and its components for mortality at 90 days, 30 days, and 1 year was assessed using time‐dependent receiver operating characteristic (ROC) curve analysis [22, 23, 24]. The time‐dependent area under the curve (AUC) at each time point was reported, along with the corresponding sensitivity, specificity, and Youden index at the optimal cut‐off point determined by maximizing the Youden index. Differences between time‐dependent AUCs were compared using the DeLong's test. To evaluate the incremental value of adding PIV to the baseline risk model, the continuous net reclassification index (NRI) and integrated discrimination improvement (IDI) were calculated. Furthermore, decision curve analysis (DCA) was employed to assess the clinical net benefit of the models by examining the balance between potential harms and benefits. The calculated effect sizes and associated P values from all the models were reported and compared. All analyses were performed with R Statistical Software (Version 4.2.2, http://www.R‐project.org, The R Foundation) and Free Statistics analysis platform (Version 2.1, Beijing, China, http://www.clinicalscientists.cn/freestatistics). A two‐sided P‐value < 0.05 was considered indicative of statistical significance.

## 3 Results

### 3.1 Baseline Characteristics of Subjects

The Baseline data are presented in Table 1. Baseline PIV levels were divided into three categories according to tertiles: T1 (< 491.1), T2 (491.4–1509.9), and T3 (≥ 1512.0). The cohort comprised 710 patients with a mean age of 68.6 ± 15.5 years, of whom 49.4% were white and 52.8% were male. Elevated PIV was associated with a higher prevalence of chronic pulmonary disease, congestive heart failure, or sepsis. Conversely, patients with renal disease had lower PIV levels. Elevated PIV levels in patients with AIS exhibited significantly higher ACM rates at 90‐day (43.5% vs. 21.5%), 30‐day (34.6% vs. 14.8%), and 1‐year intervals (51.1% vs. 29.1%); all demonstrating statistically significant gradients (*p* < 0.001).

**TABLE 1:** Basic characteristics and outcomes of participants stratified by PIV tertiles.

Variables	Total (*n* = 710)	PIV			*p*
		T1 (< 491.1) (*n* = 237)	T2 (491.4–1509.9) (*n* = 236)	T3 (≥ 1512.0) (*n* = 237)	
Sex, male	375 (52.8)	120 (50.6)	129 (54.7)	126 (53.2)	0.675
Age (years)	68.6 ± 15.5	69.0 ± 15.1	69.1 ± 15.3	67.7 ± 16.0	0.566
Race					0.004
Asian	27 (3.8)	13 (5.5)	7 (3)	7 (3)	
White	351 (49.4)	122 (51.5)	115 (48.7)	114 (48.1)	
Black	79 (11.1)	35 (14.8)	30 (12.7)	14 (5.9)	
Others	253 (35.6)	67 (28.3)	84 (35.6)	102 (43)	
Congestive heart failure	191 (26.9)	53 (22.4)	61 (25.8)	77 (32.5)	0.041
Chronic pulmonary disease	102 (14.4)	32 (13.5)	33 (14)	37 (15.6)	0.79
Diabetes mellitus	254 (35.8)	84 (35.4)	86 (36.4)	84 (35.4)	0.966
Sepsis	386 (54.4)	108 (45.6)	120 (50.8)	158 (66.7)	< 0.001
Liver disease	38 (5.4)	12 (5.1)	9 (3.8)	17 (7.2)	0.26
Renal disease	154 (21.7)	59 (24.9)	50 (21.2)	45 (19)	0.288
Malignant cancer	66 (9.3)	24 (10.1)	17 (7.2)	25 (10.5)	0.394
Heart rate (beats/min)	81.7 ± 15.9	80.5 ± 15.9	79.9 ± 15.4	84.8 ± 16.1	0.001
SBP (mmHg)	127.9 ± 19.1	125.6 ± 19.3	130.7 ± 19.7	127.4 ± 18.0	0.013
DBP (mmHg)	69.3 ± 13.2	67.6 ± 13.8	71.3 ± 12.9	69.1 ± 12.8	0.009
MBP (mmHg)	86.3 ± 13.3	84.7 ± 13.6	88.3 ± 13.0	85.9 ± 13.1	0.012
Respiratory rate (beats/min)	19.8 ± 3.6	19.4 ± 3.7	19.3 ± 3.3	20.6 ± 3.7	< 0.001
Temperature (°C)	37.0 ± 0.5	36.9 ± 0.4	37.0 ± 0.4	37.0 ± 0.5	0.109
SpO_2_ (%)	97.0 ± 1.9	97.2 ± 1.9	97.0 ± 1.8	96.9 ± 2.2	0.244
WBC (k/ul)	11.2 (8.5, 14.5)	8.6 (6.8, 11.9)	10.8 (8.8, 13.3)	14.0 (11.5, 18.2)	< 0.001
HGB (g/dL)	11.3 ± 2.4	10.8 ± 2.2	11.8 ± 2.3	11.4 ± 2.4	< 0.001
Glucose (mg/dL)	141.0 ± 47.4	136.9 ± 46.3	137.4 ± 49.1	148.8 ± 46.0	0.009
BUN (mg/dL)	18.5 (13.0, 28.0)	18.5 (12.5, 30.0)	18.0 (12.5, 26.1)	19.0 (14.0, 29.0)	0.173
Creatinine (mg/dL)	1.0 (0.8, 1.4)	1.0 (0.8, 1.4)	1.0 (0.8, 1.4)	1.0 (0.8, 1.4)	0.868
Neutrophils (k/ul)	8.2 (6.0, 12.1)	5.4 (3.8, 7.4)	7.8 (6.5, 10.0)	12.7 (10.1, 16.2)	< 0.001
Lymphocytes (k/ul)	1.3 (0.8, 1.9)	1.5 (1.0, 2.2)	1.4 (0.9, 1.9)	1.1 (0.7, 1.5)	< 0.001
Monocytes (k/ul)	0.7 (0.5, 1.1)	0.4 (0.2, 0.7)	0.8 (0.6, 1.0)	1.1 (0.8, 1.4)	< 0.001
Platelets (k/ul)	202.1 ± 90.6	147.1 ± 67.9	206.1 ± 75.2	253.2 ± 93.4	< 0.001
PIV	872.0 (353.2, 2009.8)	234.7 (92.9, 353.0)	872.0 (682.7, 1153.2)	2799.7 (2011.6, 4241.0)	< 0.001
SAPS II	37.7 ± 13.9	38.6 ± 15.1	35.5 ± 12.9	38.9 ± 13.5	0.013
APS III	52.4 ± 25.7	53.2 ± 29.1	48.5 ± 23.2	55.4 ± 23.9	0.011
Vasoactive agents	220 (31.0)	77 (32.5)	68 (28.8)	75 (31.6)	0.664
Mechanical ventilation	350 (49.3)	121 (51.1)	104 (44.1)	125 (52.7)	0.135
90‐day ACM	228 (32.1)	51 (21.5)	74 (31.4)	103 (43.5)	< 0.001
30‐day ACM	163 (23.0)	35 (14.8)	46 (19.5)	82 (34.6)	< 0.001
1‐year ACM	277 (39.0)	69 (29.1)	87 (36.9)	121 (51.1)	< 0.001

*Note:* Continuous variables were reported as the mean (standard deviation) or median (interquartile range), and categorical variables as numbers (percentages).

Abbreviations: ACM, all‐cause mortality; APS III, acute physiology score III; BUN, blood urea nitrogen; DBP, diastolic blood pressure; HGB, hemoglobin; MBP, mean blood pressure; PIV, pan‐immune‐inflammation value; SAPS II, simplified acute physiology score II; SBP, systolic blood pressure; SpO_2_, peripheral capillary oxygen saturation; T, tertile; WBC, white blood cell count.

### 3.2 Survival Analysis

**FIGURE 1:**
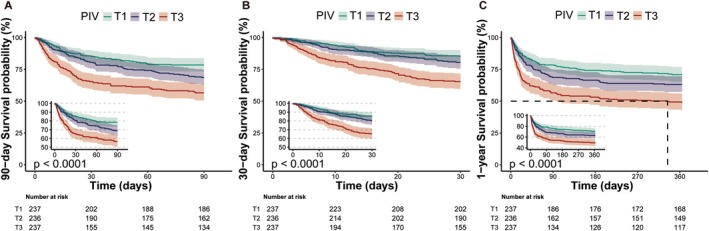
The Kaplan–Meier survival analysis of 90‐day (A), 30‐day (B), and 1‐year (C) ACM. ACM, all‐cause mortality; AIS, acute ischemic stroke; ICU, intensive care unit; PIV, pan‐immune‐inflammation value; T, tertile.

For patients with AIS who underwent follow‐up observation, the cumulative hazard of ACM at 90 days, 30 days, and 1 year was higher in individuals with higher PIV, as illustrated by the K–M curves (all *p* for log‐rank test < 0.0001; Figure 1).

### 3.3 Association Between PIV and ACM

The results of the univariate Cox regression are presented in Supplemental Table 3, Supplemental Table 4, and Supplemental Table 5. Subsequently, multivariate analysis results are presented in Table 2. In the multivariable Cox proportional hazards model, after adjusting for all potential confounders, PIV, expressed as a continuous variable (per 1000 units), was positively associated with 90‐day (HR = 1.08; 95% CI 1.03–1.13; *p* = 0.001), 30‐day (HR = 1.1; 95% CI 1.05–1.16; *p* < 0.001), and 1‐year (HR = 1.08; 95% CI 1.04–1.13; *p* < 0.001) ACM risk (Table 2, Model 3). These results demonstrate that a 1000‐unit increment in PIV increases the ACM at 90 days, 30 days, and 1 year by 8.0%, 10.0%, and 8.0%, respectively (Table 2, Model 3). Further analysis revealed that the multivariable‐adjusted HRs for 90‐day, 30‐day, and 1‐year ACM associated with PIV in T3 group were 2.7 (95% CI 1.74–4.18; *p* < 0.001), 2.5 (95% CI 1.72–3.36; *p* < 0.001), and 2.27 (95% CI 1.63–3.16; *p* < 0.001), respectively, when compared to the T1 group (Table 2, Model 3). The general trend for the association between PIV and the 90‐day, 30‐day, and 1‐year ACM was statistically significant (*p* < 0.001), as ascertained by the trend test (Table 2). All models exhibited statistically significant trends (*p* < 0.001), confirming stable positive associations.

**TABLE 2:** Multivariate Cox regression analysis between PIV and 90‐day, 30‐day, and 1‐year ACM.

	Model 1		Model 2		Model 3	
	HR (95% CI)	*P*	HR (95% CI)	*P*	HR (95% CI)	*P*
90‐day ACM						
PIV per 1000 units	1.12 (1.08–1.16)	< 0.001	1.12 (1.08–1.16)	< 0.001	1.08 (1.03–1.13)	0.001
PIV tertile						
T1 (< 491.1)	1 (Ref)		1 (Ref)		1 (Ref)	
T2 (491.4–1509.9)	1.51 (1.06–2.16)	0.023	1.53 (1.07–2.18)	0.021	1.78 (1.22–2.60)	0.003
T3 (≥ 1512.0)	2.42 (1.73–3.38)	< 0.001	2.54 (1.81–3.57)	< 0.001	2.50 (1.72–3.63)	< 0.001
*p* for trend		< 0.001		< 0.001		< 0.001
30‐day ACM						
PIV per 1000 units	1.13 (1.09–1.18)	< 0.001	1.13 (1.09–1.18)	< 0.001	1.10 (1.05–1.16)	< 0.001
PIV tertile						
T1 (< 491.1)	1 (Ref)		1 (Ref)		1 (Ref)	
T2 (491.4–1509.9)	1.35 (0.87–2.09)	0.183	1.32 (0.85–2.05)	0.218	1.59 (0.99–2.53)	0.054
T3 (≥ 1512.0)	2.69 (1.81–3.99)	< 0.001	2.64 (1.77–3.95)	< 0.001	2.70 (1.74–4.18)	< 0.001
*p* for trend		< 0.001		< 0.001		< 0.001
1‐year ACM						
PIV per 1000 units	1.12 (1.08–1.16)	< 0.001	1.12 (1.08–1.16)	< 0.001	1.08 (1.04–1.13)	< 0.001
PIV tertile						
T1 (< 491.1)	1 (Ref)		1 (Ref)		1 (Ref)	
T2 (491.4–1509.9)	1.34 (0.97–1.83)	0.072	1.36 (0.99–1.86)	0.061	1.55 (1.11–2.17)	0.011
T3 (≥ 1512.0)	2.16 (1.61–2.90)	< 0.001	2.33 (1.72–3.14)	< 0.001	2.27 (1.63–3.16)	< 0.001
*p* for trend		< 0.001		< 0.001		< 0.001

*Note:* Model 1: adjusted for none. Model 2: adjusted for age, sex, race. Model 3: adjusted for age, sex, race, congestive heart failure, diabetes mellitus, chronic pulmonary disease, sepsis, liver disease, renal disease, malignant cancer, heart rate, SBP, DBP, MBP, temperature, respiratory rate, SpO_2_, WBC, HGB, glucose, creatinine, BUN, SAPS II, APS III, vasoactive agents, mechanical ventilation.

Abbreviations: ACM, all‐cause mortality; APS III, acute physiology score III; BUN, blood urea nitrogen; CI, confidence interval; DBP, diastolic blood pressure; HGB, haemoglobin; HR, hazard ratio; MBP, mean blood pressure; PIV, pan‐immune‐inflammation value; Ref, reference; SAPS II, simplified acute physiology score II; SBP, systolic blood pressure; SpO_2_, peripheral capillary oxygen saturation; T, tertile; WBC, white blood cell count.

### 3.4 A Reverse L‐Shaped Association and Threshold Effect Between PIV and ACM

**FIGURE 2:**
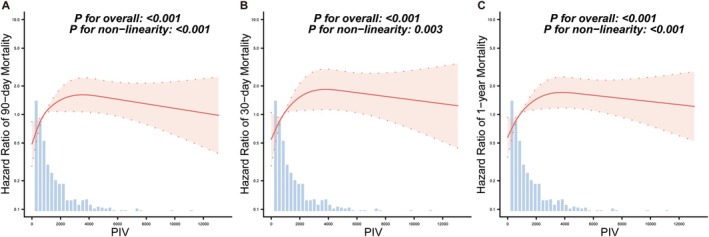
Non‐linear dose–response relationship between PIV and 90‐day (A), 30‐day (B), and 1‐year (C) ACM. Analyses employed a covariate‐adjusted Cox proportional hazards regression model incorporating restricted cubic splines with 4 knots. PIV was analyzed as a continuous variable. Curve fitted for 0%–99% of data. The red curve lines indicated the estimated values, and the surrounding shaded ribbons indicated their associated 95% confidence intervals. The analyses were adjusted for age, sex, race, congestive heart failure, diabetes mellitus, chronic pulmonary disease, sepsis, liver disease, renal disease, malignant cancer, heart rate, SBP, DBP, MBP, temperature, respiratory rate, SpO_2_, WBC, HGB, glucose, creatinine, BUN, SAPS II, APS III, vasoactive agents, mechanical ventilation. ACM, all‐cause mortality; AIS, acute ischemic stroke; APS III, acute physiology score III; BUN, blood urea nitrogen; DBP, diastolic blood pressure; HGB, hemoglobin; ICU, intensive care unit; MBP, mean blood pressure; PIV, pan‐immune‐inflammation value; SAPS II, simplified acute physiology score II; SBP, systolic blood pressure; SpO_2_, peripheral capillary oxygen saturation; WBC, white blood cell count.

In the RCS analysis, after adjusting for multiple confounders and visualizing data from 0% to 99%, a reverse L‐shaped association was observed between PIV and 90‐day mortality in ICU patients with AIS (*p* for nonlinearity < 0.001; Figure 2A). Subsequent analysis employing a two‐piecewise linear regression model identified a threshold PIV value of 2987.61 (Supplemental Table 6). Below this inflection point, PIV exhibited a significant positive correlation with the 90‐day ACM (HR = 1.491; 95% CI 1.209–1.838; *p* < 0.001) (Supplemental Table 6). After PIV up to the turning point, the estimated dose–response curve was consistent with a horizontal line, and no statistically significant association was observed between PIV and 90‐day ACM (*p* = 0.1359) (Supplemental Table 6). Similar non‐linear relationships and threshold values between PIV and 30‐day and 1‐year ACM were also observed (Figure 2B,C; Supplemental Table 6). When patients were stratified based on the PIV threshold of 2987.61, 58 patients (52.7%) in the high‐PIV group experienced the 90‐day ACM, whereas 170 of the 600 patients (28.3%) in the low‐PIV group did (Table 3). The absolute difference in the 90‐day ACM risk between the two groups was 24.4% (*p* < 0.001, Table 3). Consistent results were observed at the other endpoints (all *p* < 0.001, Table 3).

**TABLE 3:** Absolute event rates above and below the threshold of PIV at the 90‐day, 30‐day, and 1‐year ACM.

Groups	Events/N	Absolute event rates	*P*
90‐day ACM			
Low‐PIV group (< 2987.61)	170/600	28.33%	< 0.001
High‐PIV group (≥ 2987.61)	58/110	52.73%	
30‐day ACM			
Low‐PIV group (< 2696.02)	114/587	19.42%	< 0.001
High‐PIV group (≥ 2696.02)	49/123	39.84%	
1‐year ACM			
Low‐PIV group (< 3478.76)	221/620	35.65%	< 0.001
High‐PIV group (≥ 3478.76)	56/90	62.22%	

Abbreviations: ACM, all‐cause mortality; PIV, pan‐immune‐inflammation value.

### 3.5 Subgroup Analysis

**FIGURE 3:**
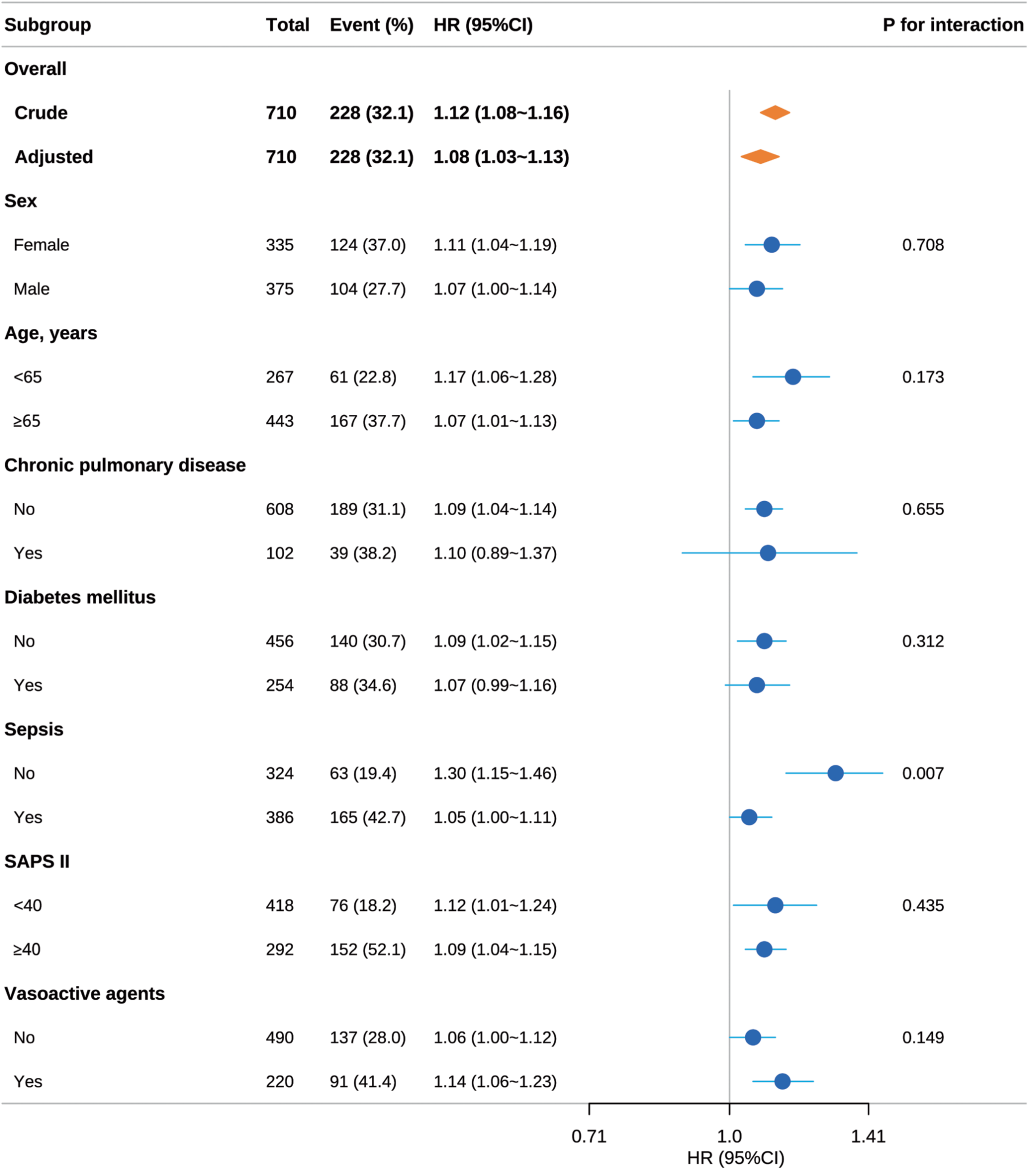
Subgroup analysis for the effect between PIV and 90‐day ACM. PIV was expressed as a continuous variable (per 1000 units). Each stratification factor was adjusted for sex, age, race, congestive heart failure, chronic pulmonary disease, diabetes mellitus, sepsis, liver disease, renal disease, malignant cancer, heart rate, SBP, DBP, MBP, respiratory rate, temperature, SpO_2_, WBC, HGB, glucose, BUN, creatinine, SAPS II, APS III, vasoactive agents and mechanical ventilation. If the stratification factor was a categorical variable, it was omitted from the subgroup analysis. ACM, all‐cause mortality; AIS, acute ischemic stroke; APS III, acute physiology score III; BUN, blood urea nitrogen; CI, confidence interval; DBP, diastolic blood pressure; HGB, hemoglobin; HR, hazard ratio; ICU, intensive care unit; MBP, mean blood pressure; PIV, pan‐immune‐inflammation value; SAPS II, simplified acute physiology score II; SBP, systolic blood pressure; SpO2, peripheral capillary oxygen saturation; WBC, white blood cell count.

The subgroup analyses of the 90‐day survival endpoint are presented in Figure 3. A statistically significant interaction was observed only in the sepsis subgroup (*p* for interaction = 0.007). The association between PIV and 90‐day ACM risk was markedly stronger in patients without sepsis (adjusted HR = 1.30, 95% CI 1.15–1.46) than in those with sepsis (adjusted HR = 1.05, 95% CI 1.00–1.11). No significant effect modification was detected across the other stratified groups (all *p* for interaction > 0.05). Consistent interaction patterns were observed for 30‐day and 1‐year survival endpoints (Supplemental Figures 2 and 3).

### 3.6 Sensitivity Analysis

After removing patients with missing values, sensitivity analysis showed that the relationship between PIV levels and 90‐day ACM remained stable (Supplemental Table 7). The results from the multiple‐imputation analyses were consistent with the primary findings, indicating that the associations between PIV and the risk of outcomes remained stable (Supplemental Table 8).

### 3.7 Prognostic Value of PIV as a Standalone Predictor

Overall, PIV showed a time‐dependent AUC of 0.6205 (95% CI: 0.5751–0.6660) for 90‐day mortality, which was numerically higher than its individual components (Supplemental Table 9 and Supplemental Figure 4). Similar patterns were observed at 30 days and 1 year. The Youden index for PIV at 90 days was 0.2125, indicating an optimal balance between sensitivity and specificity.

### 3.8 Incremental Predictive Value of PIV on a Baseline Clinical Model

When added to the baseline clinical model, PIV did not significantly improve the predictive performance. Indeed, for 90‐day ACM, the increase in the C‐statistic was not significant (0.812; *p* = 0.097), and improvements in the NRI (0.012; *p* = 0.495) and IDI (0.011; *p* = 0.08) were also not significant (Supplemental Figure 5 and Table 10). This absence of significant incremental effect was consistent with the other endpoints. DCA confirmed that the model with PIV offered no additional net benefit over the baseline risk model across the relevant decision thresholds (Supplemental Figure 6).

## 4 Discussion

The findings of this study establish the dual role of PIV as both a powerful prognostic biomarker and clinical stratification tool in critically ill patients with AIS. In its role as a biomarker, PIV was not only significantly associated with short‐ and long‐term mortality but also exhibited a reverse L‐shaped relationship with mortality risk. This characterization enables its second role: risk stratification. PIV demonstrated superior predictive performance as a single marker over its individual components and, more importantly, enabled effective risk stratification by identifying patients with significantly elevated absolute risk of death based on a clinically relevant threshold.

An important finding of this study was the non‐linear L‐shaped association between PIV and ACM in AIS patients. The steep rise in mortality risk at lower PIV values indicates that even modest elevations in systemic inflammation may be harmful. However, the plateau effect observed beyond the corresponding threshold may reflect a saturation point, at which the pathophysiological impact of inflammation is maximal. Once this level is reached, the mortality risk may be driven further by other factors, such as irreversible organ injury, severe comorbidities, or underlying frailty. Therapeutically, this pattern suggests that anti‐inflammatory interventions may achieve the greatest benefit when initiated before patients cross the saturation threshold, highlighting a potential window for timely immunomodulatory management. Subgroup analyses further showed that the association was markedly stronger in patients without sepsis than in those with sepsis, indicating that PIV is not merely a marker of infection, but also a broader indicator of immune–inflammatory dysregulation capable of identifying high‐risk patients regardless of infectious status.

Notably, PIV did not significantly enhance the baseline risk model in this study, although it outperformed each of its individual components when evaluated as a standalone marker. This is likely because the robust baseline risk model already integrates key inflammatory proxies, leaving little room for additional signals from PIV. Another finding of this study was that the absolute mortality rate was significantly higher in the high‐PIV group than in the low‐PIV group when stratified by the corresponding PIV threshold. These results reinforce principal advantage of PIV: it serves as a simple and powerful standalone stratifier, rather than a mere component of a complex score. Further, the calculation of PIV from a single blood test precludes the need to collect numerous variables, making it an ideal tool for swift risk assessment in time‐sensitive or resource‐constrained clinical environments.

Increasing evidence indicates that the immune system and inflammation are closely associated with stroke and play dual roles in its stroke pathophysiology [3, 4, 25]. Post‐stroke immunosuppression results from complex and multi‐mechanistic interactions involving extensive dysregulation of the neuro–immune–endocrine network. Pathophysiological processes following stroke exhibit temporal and spatial variability [2].

In terms of the temporal profile, the immune system shows markedly different manifestations in the acute and chronic phases after stroke. In the acute phase, a neuroinflammatory process begins in the brain immediately post‐stroke, triggering systemic immunodepression mainly through excessive activation of the autonomic nervous system, abnormal neuroendocrine regulation manifested by stimulation of the hypothalamic–pituitary–adrenal (HPA) axis, dysfunction of immune cells, release of danger‐associated molecular patterns (DAMPs) and inflammatory mediators, and gut microbiota dysbiosis [2, 3, 4, 26, 27, 28]. Systemic immunodepression, also known as stroke‐induced immunodepression syndrome (SIDS), promotes various infections [29, 30]. The most common infectious complications are stroke‐associated pneumonia (SAP) and urinary tract infections [4, 31]. Nevertheless, there are also beneficial protective effects on the immunosuppression process. In the chronic phase, long‐term neurological deficits after stroke, such as cognitive decline, dementia, depression, and fatigue, may be associated with global brain inflammation [2]; however, many aspects of its initiation and progression remain unclear.

In terms of spatial distribution, stroke not only affects the focal region of brain injury, but rather has widespread effects on the entire brain [2]. Stroke results in neuronal cell death and the subsequent release of DAMPs, triggering localized inflammation in the affected brain region. This focal inflammatory response contributes to secondary brain injury by exacerbating blood–brain barrier (BBB) damage, microvascular injury, brain edema, oxidative stress, and directly inducing neuronal cell death. Beyond the site of the injured brain, growing evidence indicates that post‐stroke inflammation extends throughout the brain and persists over time. Global brain inflammation may continuously shape disease progression after a stroke and ultimately influence long‐term neurological outcomes. In addition, the detrimental consequences of stroke extend beyond the brain, affecting the cardiac, endocrine, gastrointestinal, lymphoid, and musculoskeletal tissues. This highlights the bidirectional crosstalk between the brain and these organ systems and supports the characterization of stroke as a systemic disorder rather than solely a neurological condition [5].

Therefore, it could be concluded that immunity and inflammation are involved throughout the AIS process and have long‐term effects on AIS. Despite the limited amount of research on PIV in the context of cardiovascular and cerebrovascular diseases, existing studies have suggested that PIV may possess potential predictive value for these conditions because of its ability to assess systemic inflammation. In the present study, PIV was identified as a significant independent prognostic marker in critically ill patients with AIS. This marker integrates immune–inflammatory information more effectively than any single component and distills this complexity into a practical, actionable tool for risk stratification capable of pinpointing patients at a substantially heightened risk of mortality.

Overall, this study provides important evidence of the prognostic value of PIV for both early and late ACM in ICU patients with critical AIS, as analyzed using the MIMIC‐IV database. The major strengths of this study are the large sample size, coverage, and representativeness of the US population, which provided sufficient power to examine the findings. Moreover, the research perspective adopted in the present study is genuine and clinically translatable. These findings provide an essential foundation for the development of PIV‐guided mortality risk stratification and mechanistic exploration of immune and inflammatory pathways in cerebrovascular critical care.

However, this study had several limitations that need to be considered. First, although we rigorously adjusted for multiple covariates using multivariate regression models, the retrospective observational design inherently limited causal inference. Second, residual confounding from unmeasured variables (e.g., socioeconomic status, nutritional profiles, or genetic predisposition) could potentially influence ACM risk estimates. Third, the study design restricted PIV assessment to baseline measurements, thus precluding the evaluation of temporal variations during the critical care phases (hospitalization and ICU stay) and post‐discharge periods. Future prospective investigations incorporating serial biomarker measurements are warranted to characterize the dynamic trajectories of PIV and their prognostic implications. Fourth, the generalizability of our conclusions requires validation, as the exclusively US‐based cohort may not fully represent the pathophysiological patterns in global populations. Given these limitations, well‐designed large‐scale multicenter trials are essential to verify our findings, complemented by mechanistic investigations to elucidate the biological underpinnings of PIV‐mortality associations.

## 5 Conclusions

Overall, this cohort study identified a reverse L‐shaped relationship between PIV and short‐ and long‐term ACM in ICU‐admitted patients with AIS. PIV can serve as a cost‐effective and clinically practical tool for the early identification of patients with AIS at a high risk of adverse outcomes.

## Author Contributions

Zhenzhu Li: conceptualization, data curation, methodology, funding acquisition, and writing.

## Funding

This work was supported by Tianjin Union Medical Center, 2019YJ012. Tianjin Municipal Health Commission for Public Health Affairs, 2023058.

## Ethics Statement

MIMIC‐IV database used in this study was approved by the IRBs of the Beth Israel Deaconess Medical Center (2001‐P‐001699/14) and the Massachusetts Institute of Technology (No. 0403000206).

## Consent

The requirement for informed consent was waived, given that all information in MIMIC‐IV is anonymized.

## Conflicts of Interest

The author declares no conflicts of interest.

## Supporting information


**Table S1:** The detailed ICD codes of ischemic stroke in MIMIC‐IV database.
**Table S2:** The proportion of missing values for the extracted variables.
**Table S3:** Univariate Cox regression analysis for 90‐day ACM.
**Table S4:** Univariate Cox regression analysis for 30‐day ACM.
**Table S5:** Univariate Cox regression analysis for 1‐year ACM.
**Table S6:** Threshold effect analysis of the association between PIV with 90‐day, 30‐day, and 1‐year ACM.
**Table S7:** Multivariate Cox regression analysis of patients with missing values excluded.
**Table S8:** Multivariate Cox regression analysis after multiple imputation of missing values.
**Table S9:** Predictive performance of PIV and its components for 90‐day, 30‐day, and 1‐year ACM assessed by time‐dependent receiver operating characteristic analysis.
**Table S10:** Incremental predictive value of PIV for 90‐day, 30‐day, and 1‐year ACM assessed by C‐statistic, NRI, and IDI.
**Figure S1:** Flow chart of study population selection.
**Figure S2:** Subgroup analysis for the effect between PIV and 30‐day ACM.
**Figure S3:** Subgroup analysis for the effect between PIV and 1‐year ACM.
**Figure S4:** Time‐dependent receiver operating characteristic curves of PIV and its components for 90‐day (A), 30‐day (B), and 1‐year (C) ACM in critically ill AIS patients.
**Figure S5:** Time‐dependent receiver operating characteristic curves of the models with and without PIV for 90‐day (A), 30‐day (B), and 1‐year (C) ACM in critically ill AIS patients.
**Figure S6:** Decision curve analysis of the models with and without PIV for 90‐day (A), 30‐day (B), and 1‐year (C) ACM in critically ill AIS patients.

## Data Availability

The data utilized in this study can be obtained from the MIMIC‐IV v3.1 database (https://physionet.org/content/mimiciv/3.1/). The datasets generated and analyzed in this study are available from the corresponding author upon reasonable request.
