# Supplementary material for: Pan‐Immune‐Inflammation Value for Mortality Risk Stratification in Critically Ill Patients With Acute Ischemic Stroke: A Cohort Study Based on the MIMIC‐IV Database
Source: CNS Neurosci Ther. 2026 Mar 13;32(3):e70831. doi: 10.1002/cns.70831 (PMC13093308; doi:10.1002/cns.70831)
Supplement: Supplementary file 1 — Table S1: The detailed ICD codes of ischemic stroke in MIMIC‐IV database. Table S2: The proportion of missing values for the extracted variables. Table S3: Univariate Cox regression analysis for 90‐day ACM. Table S4: Univariate Cox regression analysis for 30‐day ACM. Table S5: Univariate Cox regression analysis for 1‐year ACM. Table S6: Threshold effect analysis of the association between PIV with 90‐day, 30‐day, and 1‐year ACM. Table S7: Multivariate Cox regression analysis of patients with missing values excluded. Table S8: Multivariate Cox regression analysis after multiple imputation of missing values. Table S9: Predictive performance of PIV and its components for 90‐day, 30‐day, and 1‐year ACM assessed by time‐dependent receiver operating characteristic analysis. Table S10: Incremental predictive value of PIV for 90‐day, 30‐day, and 1‐year ACM assessed by C‐statistic, NRI, and IDI. Figure S1: Flow chart of study population selection. Figure S2: Subgroup analysis for the effect between PIV and 30‐day ACM. Figure S3: Subgroup analysis for the effect between PIV and 1‐year ACM. Figure S4: Time‐dependent receiver operating characteristic curves of PIV and its components for 90‐day (A), 30‐day (B), and 1‐year (C) ACM in critically ill AIS patients. Figure S5: Time‐dependent receiver operating characteristic curves of the models with and without PIV for 90‐day (A), 30‐day (B), and 1‐year (C) ACM in critically ill AIS patients. Figure S6: Decision curve analysis of the models with and without PIV for 90‐day (A), 30‐day (B), and 1‐year (C) ACM in critically ill AIS patients. [file CNS-32-e70831-s001.docx]

**Supplemental materials**

Supplemental Table 1 The detailed ICD codes of ischemic stroke in MIMIC-IV database.

Supplemental Table 2 The proportion of missing values for the extracted variables.

Supplemental Table 3 Univariate Cox regression analysis for 90-day ACM.

Supplemental Table 4 Univariate Cox regression analysis for 30-day ACM.

Supplemental Table 5 Univariate Cox regression analysis for 1-year ACM.

Supplemental Table 6 Threshold effect analysis of the association between PIV with 90-day, 30-day, and 1-year ACM.

Supplemental Table 7 Multivariate Cox regression analysis of patients with missing values excluded.

Supplemental Table 8 Multivariate Cox regression analysis after multiple imputation of missing values

Supplemental Table 9 Predictive performance of PIV and its components for 90-day, 30-day, and 1-year ACM assessed by time-dependent receiver operating characteristic analysis.

Supplemental Table 10 Incremental predictive value of PIV for 90-day, 30-day, and 1-year ACM assessed by C-statistic, NRI, and IDI

Supplemental Figure 1 Flow chart of study population selection.

Supplemental Figure 2 Subgroup analysis for the effect between PIV and 30-day ACM.

Supplemental Figure 3 Subgroup analysis for the effect between PIV and 1-year ACM.

Supplemental Figure 4 Time-dependent receiver operating characteristic curves of PIV and its components for 90-day (A), 30-day (B), and 1-year (C) ACM in critically ill AIS patients.

Supplemental Figure 5 Time-dependent receiver operating characteristic curves of the models with and without PIV for 90-day (A), 30-day (B), and 1-year (C) ACM in critically ill AIS patients.

Supplemental Figure 6 Decision curve analysis of the models with and without PIV for 90-day (A), 30-day (B), and 1-year (C) ACM in critically ill AIS patients.

**Supplemental Table 1** The detailed ICD codes of ischemic stroke in MIMIC-IV database.

| **Items** | **Composition** |
| --- | --- |
| ICD-9 codes | 43, 43301, 43311, 43321, 43331, 43381, 43391, 43401, 43411 |
| ICD-10 codes | I63, I6300, I6302, I6309, I63011 to I63013, I63031, I63032, I636, I638, I6310, I6312, I6319, I6320, I6322, I6329, I6330, I6339, I6340, I6349, I6350, I6359, I6381, I6389, I63111 to I63113, I63119, I63131 to I63133, I63139, I63211 to I63213, I63219, I63231 to I63233, I63239, I63311 to I63313, I63321, I63322, I63331 to I63333, I63341 to I63343, I63349, I63411 to I63413, I63419, I63421 to I63423, I63429, I63431 to I63433, I63439, I63441 to I63443, I63449, I63511 to I63513, I63521 to I63523, I63531 to I63533, I63539, I63541 to I63543, I63549 |

**Supplemental Table 2** The proportion of missing values for the extracted variables.

| **Variables** | **Missing data (n)** | **Missing data (%)** |
| --- | --- | --- |
| Age | 0 |  |
| Sex | 0 |  |
| Race | 0 |  |
| Congestive heart failure | 0 |  |
| Chronic pulmonary disease | 0 |  |
| Diabetes mellitus | 0 |  |
| Sepsis | 0 |  |
| Liver disease | 0 |  |
| Renal disease | 0 |  |
| Malignant cancer | 0 |  |
| Heart rate | 0 |  |
| SBP | 7 | <1% |
| DBP | 7 | <1% |
| MBP | 0 |  |
| Respiratory rate | 0 |  |
| Temperature | 16 | 2.20% |
| SpO_2_ | 0 |  |
| WBC | 6 | <1% |
| HGB | 6 | <1% |
| Glucose | 2 | <1% |
| BUN | 6 | <1% |
| Creatinine | 7 | <1% |
| SAPS II | 0 |  |
| APS III | 0 |  |
| Vasoactive agents | 0 |  |
| Mechanical ventilation | 0 |  |
| 90-day mortality | 0 |  |
| 30-day mortality | 0 |  |
| 1-year mortality | 0 |  |

PIV, pan-immune-inflammation value; SBP, systolic blood pressure; DBP, diastolic blood pressure; MAP, mean blood pressure; SpO_2_, peripheral capillary oxygen saturation; WBC, white blood cell count; HGB, hemoglobin; BUN, blood urea nitrogen; SAPS II, simplified acute physiology score II; APS III, acute physiology score III.

**Supplemental Table 3** Univariate Cox regression analysis for 90-day ACM.

| **Variables** | **HR (95% CI)** | ***P* value** |
| --- | --- | --- |
| Sex | 0.7 (0.54,0.9) | 0.006 |
| Age | 1.03 (1.02,1.04) | < 0.001 |
| Race |  |  |
| White | 1.02 (0.49,2.08) | 0.967 |
| Black | 1.0081 (0.4529,2.244) | 0.984 |
| Others | 1.35 (0.65,2.78) | 0.418 |
| Congestive heart failure | 1.67 (1.27,2.18) | < 0.001 |
| Chronic pulmonary disease | 1.29 (0.91,1.82) | 0.152 |
| Diabetes mellitus | 1.15 (0.88,1.5) | 0.3 |
| Sepsis | 2.54 (1.9,3.4) | < 0.001 |
| Liver disease | 1.43 (0.86,2.38) | 0.165 |
| Renal disease | 1.66 (1.25,2.21) | < 0.001 |
| Malignant cancer | 2.05 (1.43,2.92) | < 0.001 |
| Heart rate | 1.02 (1.01,1.02) | < 0.001 |
| SBP | 0.9944 (0.9875,1.0013) | 0.113 |
| DBP | 0.98 (0.97,0.99) | 0.002 |
| MBP | 0.99 (0.98,1) | 0.013 |
| Respiratory rate | 1.09 (1.05,1.12) | < 0.001 |
| Temperature | 1.42 (1.05,1.92) | 0.023 |
| SpO_2_ | 0.9941 (0.9299,1.0627) | 0.863 |
| WBC | 1.01 (1,1.02) | 0.019 |
| HGB | 0.92 (0.87,0.97) | 0.003 |
| Glucose | 1.004 (1.0018,1.0062) | < 0.001 |
| BUN | 1.01 (1.01,1.02) | < 0.001 |
| Creatinine | 1.09 (1.03,1.15) | 0.002 |
| Neutrophils | 1.03 (1.02,1.05) | < 0.001 |
| Lymphocytes | 0.95 (0.87,1.04) | 0.297 |
| Monocytes | 1.11 (1.04,1.19) | 0.003 |
| Platelets | 0.9998 (0.9983,1.0013) | 0.803 |
| PIV per 1000 units | 1.12 (1.08,1.16) | < 0.001 |
| SAPS II | 1.05 (1.04,1.05) | < 0.001 |
| APS III | 1.02 (1.01,1.02) | < 0.001 |
| Vasoactive agents | 1.66 (1.27,2.17) | < 0.001 |
| Mechanical ventilation | 2.14 (1.63,2.8) | < 0.001 |

PIV, pan-immune-inflammation value; ACM, all-cause mortality; HR, hazard ratio; SBP, systolic blood pressure; DBP, diastolic blood pressure; MAP, mean blood pressure; SpO_2_, peripheral capillary oxygen saturation; WBC, white blood cell count; HGB, hemoglobin; BUN, blood urea nitrogen; SAPS II, simplified acute physiology score II; APS III, acute physiology score III.

**Supplemental Table 4** Univariate Cox regression analysis for 30-day ACM.

| **Variables** | **HR (95% CI)** | ***P* value** |
| --- | --- | --- |
| Sex | 0.69 (0.51,0.94) | 0.018 |
| Age | 1.02 (1.01,1.03) | < 0.001 |
| Race |  |  |
| White | 1.15 (0.46,2.84) | 0.765 |
| Black | 0.73 (0.26,2.11) | 0.567 |
| Others | 1.68 (0.68,4.16) | 0.262 |
| Congestive heart failure | 1.39 (1,1.93) | 0.048 |
| Chronic pulmonary disease | 1.22 (0.8,1.84) | 0.354 |
| Diabetes mellitus | 1.14 (0.83,1.56) | 0.414 |
| Sepsis | 2.64 (1.86,3.75) | < 0.001 |
| Liver disease | 1.73 (1,2.99) | 0.05 |
| Renal disease | 1.48 (1.05,2.08) | 0.025 |
| Malignant cancer | 1.71 (1.1,2.66) | 0.017 |
| Heart rate | 1.02 (1.01,1.03) | < 0.001 |
| SBP | 0.9916 (0.9833,0.9999) | 0.048 |
| DBP | 0.98 (0.97,1) | 0.01 |
| MBP | 0.99 (0.97,1) | 0.02 |
| Respiratory rate | 1.09 (1.05,1.14) | < 0.001 |
| Temperature | 1.46 (1.03,2.08) | 0.033 |
| SpO_2_ | 0.98 (0.9,1.05) | 0.518 |
| WBC | 1.01 (1,1.02) | 0.005 |
| HGB | 0.92 (0.86,0.99) | 0.019 |
| Glucose | 1.0041 (1.0016,1.0067) | 0.002 |
| BUN | 1.01 (1.01,1.02) | < 0.001 |
| Creatinine | 1.09 (1.02,1.16) | 0.009 |
| Neutrophils | 1.04 (1.02,1.05) | < 0.001 |
| Lymphocytes | 0.98 (0.92,1.06) | 0.671 |
| Monocytes | 1.09 (1.01,1.19) | 0.032 |
| Platelets | 1.0001 (0.9984,1.0018) | 0.912 |
| PIV per 1000 units | 1.13 (1.09,1.18) | < 0.001 |
| SAPS II | 1.04 (1.03,1.05) | < 0.001 |
| APS III | 1.02 (1.02,1.03) | < 0.001 |
| Vasoactive agents | 1.79 (1.31,2.44) | < 0.001 |
| Mechanical ventilation | 2.4 (1.73,3.33) | < 0.001 |

PIV, pan-immune-inflammation value; ACM, all-cause mortality; HR, hazard ratio; SBP, systolic blood pressure; DBP, diastolic blood pressure; MAP, mean blood pressure; SpO_2_, peripheral capillary oxygen saturation; WBC, white blood cell count; HGB, hemoglobin; BUN, blood urea nitrogen; SAPS II, simplified acute physiology score II; APS III, acute physiology score III.

**Supplemental Table 5** Univariate Cox regression analysis for 1-year ACM.

| **Variables** | **HR (95% CI)** | ***P* value** |
| --- | --- | --- |
| Sex | 0.73 (0.58,0.93) | 0.01 |
| Age | 1.03 (1.02,1.04) | < 0.001 |
| Race |  |  |
| White | 1.33 (0.65,2.71) | 0.434 |
| Black | 1.28 (0.59,2.8) | 0.533 |
| Others | 1.54 (0.75,3.15) | 0.242 |
| Congestive heart failure | 1.82 (1.43,2.32) | < 0.001 |
| Chronic pulmonary disease | 1.29 (0.94,1.77) | 0.109 |
| Diabetes mellitus | 1.24 (0.97,1.58) | 0.081 |
| Sepsis | 2.49 (1.92,3.23) | < 0.001 |
| Liver disease | 1.34 (0.83,2.16) | 0.23 |
| Renal disease | 1.67 (1.29,2.16) | < 0.001 |
| Malignant cancer | 2.01 (1.44,2.81) | < 0.001 |
| Heart rate | 1.02 (1.01,1.02) | < 0.001 |
| SBP | 0.9935 (0.9872,0.9998) | 0.043 |
| DBP | 0.98 (0.97,0.99) | < 0.001 |
| MBP | 0.99 (0.98,0.99) | 0.002 |
| Respiratory rate | 1.08 (1.05,1.11) | < 0.001 |
| Temperature | 1.26 (0.96,1.67) | 0.099 |
| SpO_2_ | 0.99 (0.93,1.05) | 0.633 |
| WBC | 1.01 (1,1.02) | 0.041 |
| HGB | 0.91 (0.87,0.96) | < 0.001 |
| Glucose | 1.0046 (1.0027,1.0066) | < 0.001 |
| BUN | 1.01 (1.01,1.02) | < 0.001 |
| Creatinine | 1.08 (1.03,1.14) | 0.003 |
| Neutrophils | 1.03 (1.02,1.04) | < 0.001 |
| Lymphocytes | 0.93 (0.84,1.03) | 0.157 |
| Monocytes | 1.11 (1.04,1.19) | 0.002 |
| Platelets | 0.9998 (0.9985,1.0012) | 0.783 |
| PIV per 1000 units | 1.12 (1.08,1.16) | < 0.001 |
| SAPS II | 1.05 (1.04,1.05) | < 0.001 |
| APS III | 1.02 (1.01,1.02) | < 0.001 |
| Vasoactive agents | 1.53 (1.2,1.95) | < 0.001 |
| Mechanical ventilation | 1.78 (1.4,2.26) | < 0.001 |

PIV, pan-immune-inflammation value; ACM, all-cause mortality; HR, hazard ratio; SBP, systolic blood pressure; DBP, diastolic blood pressure; MAP, mean blood pressure; SpO_2_, peripheral capillary oxygen saturation; WBC, white blood cell count; HGB, hemoglobin; BUN, blood urea nitrogen; SAPS II, simplified acute physiology score II; APS III, acute physiology score III.

**Supplemental Table 6** Threshold effect analysis of the association between PIV with 90-day, 30-day, and 1-year ACM.

| **PIV** | **HR (95% CI)*** | ***P* value** |
| --- | --- | --- |
| **90-day ACM** |  |  |
| < 2987.61 | 1.491 (1.209,1.838) | < 0.001 |
| ≥ 2987.61 | 0.882 (0.747,1.040) | 0.1359 |
| Likelihood Ratio test |  | <0.001 |
| **30-day ACM** |  |  |
| < 2696.02 | 1.604 (1.215,2.116) | < 0.001 |
| ≥ 2696.02 | 0.978 (0.819,1.168) | 0.8084 |
| Likelihood Ratio test |  | 0.002 |
| **1-year ACM** |  |  |
| < 3478.76 | 1.420 (1.206,1.672) | < 0.001 |
| ≥ 3478.76 | 0.849 (0.700,1.030) | 0.0969 |
| Likelihood Ratio test |  | <0.001 |

PIV was entered as a continuous variable. Curve fitted for 0%-99% of data. The analyses were adjusted for age, sex, race, congestive heart failure, diabetes mellitus, chronic pulmonary disease, sepsis, liver disease, renal disease, malignant cancer, heart rate, SBP, DBP, MBP, temperature, respiratory rate, SpO_2_, WBC, HGB, glucose, creatinine, BUN, SAPS II, APS III, vasoactive agents, mechanical ventilation.

PIV, pan-immune-inflammation value; ACM, all-cause mortality; ICU, intensive care unit; AIS, acute ischemic stroke; SBP, systolic blood pressure; DBP, diastolic blood pressure; MBP, mean blood pressure; SpO_2_, peripheral capillary oxygen saturation; WBC, white blood cell count; HGB, hemoglobin; BUN, blood urea nitrogen; SAPS II, simplified acute physiology score II; APS III, acute physiology score III.

*The term “HR (95%CI)” refers to values of the hazard ratios and their corresponding 95% confidence intervals after being per 1000-transformed.

**Supplemental Table 7** Multivariate Cox regression analysis of patients with missing values excluded.

|  | **Model 1** | | **Model 2** | | **Model 3** | |
| --- | --- | --- | --- | --- | --- | --- |
|  | **HR (95% CI)** | ***P* value** | **HR (95% CI)** | ***P* value** | **HR (95% CI)** | ***P* value** |
| **90-day ACM** | | | | | | |
| PIV per 1000 units | 1.13 (1.09–1.16) | <0.001 | 1.12 (1.08–1.16) | <0.001 | 1.09 (1.05–1.14) | <0.001 |
| PIV tertile |  |  |  |  |  |  |
| T1 (< 491.1) | 1(Ref) |  | 1(Ref) |  | 1(Ref) |  |
| T2 (491.4–1509.9) | 1.45 (1.01–2.09) | 0.043 | 1.48 (1.03–2.14) | 0.035 | 1.70 (1.16–2.5) | 0.006 |
| T3 (≥ 1512.0) | 2.34 (1.67–3.29) | <0.001 | 2.50 (1.77–3.53) | <0.001 | 2.47 (1.69–3.61) | <0.001 |
| *P* for trend |  | <0.001 |  | <0.001 |  | <0.001 |
| **30-day ACM** | | | | | | |
| PIV per 1000 units | 1.14 (1.1–1.18) | <0.001 | 1.14 (1.09–1.18) | <0.001 | 1.12 (1.07–1.17) | <0.001 |
| PIV tertile | | | | | | |
| T1 (< 491.1) | 1(Ref) |  | 1(Ref) |  | 1(Ref) |  |
| T2 (491.4–1509.9) | 1.36 (0.87–2.12) | 0.181 | 1.34 (0.86–2.10) | 0.198 | 1.56 (0.97–2.50) | 0.064 |
| T3 (≥ 1512.0) | 2.64 (1.76–3.95) | <0.001 | 2.66 (1.77–4.00) | <0.001 | 2.70 (1.73–4.24) | <0.001 |
| *P* for trend |  | <0.001 |  | <0.001 |  | <0.001 |
| **1-year ACM** | | | | | | |
| PIV per 1000 units | 1.12 (1.09–1.16) | <0.001 | 1.12 (1.08–1.16) | <0.001 | 1.09 (1.05–1.13) | <0.001 |
| PIV tertile | | | | | | |
| T1 (< 491.1) | 1(Ref) |  | 1(Ref) |  | 1(Ref) |  |
| T2 (491.4–1509.9) | 1.29 (0.93–1.78) | 0.125 | 1.32 (0.96–1.83) | 0.091 | 1.50 (1.07–2.11) | 0.02 |
| T3 (≥ 1512.0) | 2.11 (1.56–2.85) | <0.001 | 2.29 (1.69–3.11) | <0.001 | 2.25 (1.60–3.16) | <0.001 |
| *P* for trend |  | <0.001 |  | <0.001 |  | <0.001 |

Model 1: adjusted for none;

Model 2: adjusted for age, sex, race;

Model 3: adjusted for age, sex, race, congestive heart failure, Diabetes mellitus, chronic pulmonary disease, sepsis, liver disease, renal disease, malignant cancer, heart rate, SBP, DBP, MBP, temperature, respiratory rate, SpO_2_, WBC, HGB, glucose, creatinine, BUN, SAPS II, APS III, vasoactive agents, mechanical ventilation.

ACM, all-cause mortality; PIV, pan-immune-inflammation value; HR, hazard ratio; CI, confidence interval; T, tertile; Ref, reference; SBP, systolic blood pressure; DBP, diastolic blood pressure; MBP, mean blood pressure; SpO_2_, peripheral capillary oxygen saturation; WBC, white blood cell count; HGB, hemoglobin; BUN, blood urea nitrogen; SAPS II, simplified acute physiology score II; APS III, acute physiology score III.

**Supplemental Table 8** Multivariate Cox regression analysis after multiple imputation of missing values

|  | **Model 1** | | **Model 2** | | **Model 3** | |
| --- | --- | --- | --- | --- | --- | --- |
|  | **HR (95% CI)** | ***P* value** | **HR (95% CI)** | ***P* value** | **HR (95% CI)** | ***P* value** |
| **90-day ACM** | | | | | | |
| PIV per 1000 units | 1.12 (1.08–1.16) | <0.001 | 1.12 (1.08–1.16) | <0.001 | 1.08 (1.03–1.13) | 0.001 |
| PIV tertile | | | | | | |
| T1 (< 491.1) | 1(Ref) |  | 1(Ref) |  | 1(Ref) |  |
| T2 (491.4–1509.9) | 1.51 (1.06–2.16) | 0.023 | 1.53 (1.07–2.18) | 0.021 | 1.78 (1.22–2.60) | 0.003 |
| T3 (≥ 1512.0) | 2.42 (1.73–3.38) | <0.001 | 2.54 (1.81–3.57) | <0.001 | 2.49 (1.72–3.62) | <0.001 |
| *P* for trend |  | <0.001 |  | <0.001 |  | <0.001 |
| **30-day ACM** | | | | | | |
| PIV per 1000 units | 1.13 (1.09–1.18) | <0.001 | 1.13 (1.09–1.18) | <0.001 | 1.1 (1.05–1.16) | <0.001 |
| PIV tertile | | | | | | |
| T1 (< 491.1) | 1(Ref) |  | 1(Ref) |  | 1(Ref) |  |
| T2 (491.4–1509.9) | 1.35 (0.87–2.09) | 0.183 | 1.32 (0.85–2.05) | 0.218 | 1.58 (0.99–2.53) | 0.055 |
| T3 (≥ 1512.0) | 2.69 (1.81–3.99) | <0.001 | 2.64 (1.77–3.95) | <0.001 | 2.69 (1.74–4.17) | <0.001 |
| *P* for trend |  | <0.001 |  | <0.001 |  | <0.001 |
| **1-year ACM** | | | | | | |
| PIV per 1000 units | 1.12 (1.08–1.16) | <0.001 | 1.12 (1.08–1.16) | <0.001 | 1.08 (1.04–1.13) | <0.001 |
| PIV tertile | | | | | | |
| T1 (< 491.1) | 1(Ref) |  | 1(Ref) |  | 1(Ref) |  |
| T2 (491.4–1509.9) | 1.34 (0.97–1.83) | 0.072 | 1.36 (0.99–1.86) | 0.061 | 1.55 (1.11–2.18) | 0.011 |
| T3 (≥ 1512.0) | 2.16 (1.61–2.90) | <0.001 | 2.33 (1.72–3.14) | <0.001 | 2.27 (1.63–3.16) | <0.001 |
| *P* for trend |  | <0.001 |  | <0.001 |  | <0.001 |

Model 1: adjusted for none;

Model 2: adjusted for age, sex, race;

Model 3: adjusted for age, sex, race, congestive heart failure, diabetes mellitus, chronic pulmonary disease, sepsis, liver disease, renal disease, malignant cancer, heart rate, SBP, DBP, MBP, temperature, respiratory rate, SpO_2_, WBC, HGB, glucose, creatinine, BUN, SAPS II, APS III, vasoactive agents, mechanical ventilation.

ACM, all-cause mortality; PIV, pan-immune-inflammation value; HR, hazard ratio; CI, confidence interval; T, tertile; Ref, reference; SBP, systolic blood pressure; DBP, diastolic blood pressure; MBP, mean blood pressure; SpO_2_, peripheral capillary oxygen saturation; WBC, white blood cell count; HGB, hemoglobin; BUN, blood urea nitrogen; SAPS II, simplified acute physiology score II; APS III, acute physiology score III.

**Supplemental Table 9** Predictive performance of PIV and its components for 90-day, 30-day, and 1-year ACM assessed by time-dependent receiver operating characteristic analysis.

| **Model** | **AUC (95% CI)** | ***P* for comparison** | **Sensitivity** | **Specificity** | **Youden index** |
| --- | --- | --- | --- | --- | --- |
| **90-day ACM** |  |  |  |  |  |
| Neutrophil | 0.6013 (0.5561–0.6464) | Ref. | 0.6009 | 0.5809 | 0.1818 |
| Monocyte | 0.5631 (0.5161–0.6101) | 0.127 | 0.5088 | 0.6307 | 0.1395 |
| Platelet | 0.5134 (0.4664–0.5604) | 0.018 | 0.1184 | 0.9523 | 0.0707 |
| Lymphocyte | 0.6092 (0.5648–0.6536) | 0.814 | 0.4912 | 0.6784 | 0.1697 |
| PIV | 0.6205 (0.5751–0.6660) | 0.302 | 0.6316 | 0.5809 | 0.2125 |
| **30-day ACM** |  |  |  |  |  |
| Neutrophil | 0.6155 (0.5645–0.6665) | Ref. | 0.6319 | 0.5777 | 0.2096 |
| Monocyte | 0.5673 (0.5150–0.6195) | 0.091 | 0.5276 | 0.6197 | 0.1474 |
| Platelet | 0.5116 (0.4587–0.5645) | 0.017 | 0.4049 | 0.6673 | 0.0722 |
| Lymphocyte | 0.6080 (0.5581–0.6579) | 0.845 | 0.5215 | 0.6673 | 0.1887 |
| PIV | 0.6311 (0.5798–0.6824) | 0.453 | 0.5521 | 0.6801 | 0.2322 |
| **1-year ACM** |  |  |  |  |  |
| Neutrophil | 0.5811 (0.5376–0.6245) | Ref. | 0.5668 | 0.5797 | 0.1465 |
| Monocyte | 0.5636 (0.5194–0.6079) | 0.46 | 0.4260 | 0.7090 | 0.1350 |
| Platelet | 0.5153 (0.4709–0.5596) | 0.063 | 0.1119 | 0.9561 | 0.0680 |
| Lymphocyte | 0.6081 (0.5657–0.6505) | 0.394 | 0.6534 | 0.5150 | 0.1684 |
| PIV | 0.6143 (0.5710–0.6577) | 0.058 | 0.6209 | 0.5751 | 0.1960 |

PIV, pan-immune-inflammation value; ACM, all-cause mortality; AIS, acute ischemic stroke; AUC, area under the curve; Cl, confidence interval.

**Supplemental Table 10** Incremental predictive value of PIV for 90-day, 30-day and 1-year ACM assessed by C-statistic, NRI, and IDI

| **Model** | **C-statistic (95% CI)** | ***P* value** | **NRI (95% CI)** | ***P* value** | **IDI (95% CI)** | ***P* value** |
| --- | --- | --- | --- | --- | --- | --- |
| **90-day ACM** |  |  |  |  |  |  |
| Baseline risk model | 0.805 (0.772–0.837) | Ref. | Ref. |  | Ref. |  |
| Baseline risk model +PIV | 0.812 (0.779–0.844) | 0.097 | 0.012 (-0.13–0.157) | 0.495 | 0.011 (-0.002–0.028) | 0.08 |
| **30-day ACM** |  |  |  |  |  |  |
| Baseline risk model | 0.794 (0.758–0.830) | Ref. | Ref. |  | Ref. |  |
| Baseline risk model +PIV | 0.805 (0.769–0.841) | 0.051 | 0.159 (-0.063–0.238) | 0.152 | 0.023 (0.002–0.051) | 0.036 |
| **1-year ACM** |  |  |  |  |  |  |
| Baseline risk model | 0.815 (0.783–0.846) | Ref. | Ref. |  | Ref. |  |
| Baseline risk model +PIV | 0.821 (0.790–0.852) | 0.104 | 0.104 (-0.1–0.201) | 0.196 | 0.011 (-0.001–0.027) | 0.064 |

Baseline risk model includes age, sex, race, congestive heart failure, diabetes mellitus, chronic pulmonary disease, sepsis, liver disease, renal disease, malignant cancer, heart rate, SBP, DBP, MBP, temperature, respiratory rate, SpO_2_, WBC, HGB, glucose, creatinine, BUN, SAPS II, APS III, vasoactive agents, mechanical ventilation. PIV, pan-immune-inflammation value; ACM, all-cause mortality; NRI, net reclassification improvement; IDI, integrated discrimination improvement; CI, confidence interval; SBP, systolic blood pressure; DBP, diastolic blood pressure; MBP, mean blood pressure; SpO_2_, peripheral capillary oxygen saturation; WBC, white blood cell count; HGB, hemoglobin; BUN, blood urea nitrogen; SAPS II, simplified acute physiology score II; APS III, acute physiology score III.

**
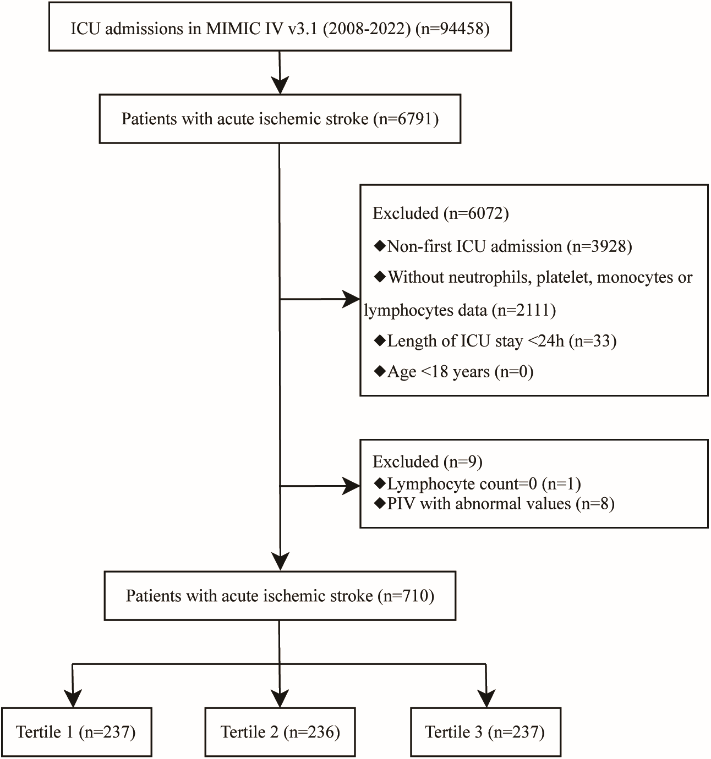
**

**Supplemental Figure 1** Flow chart of study population selection. ICU, intensive care unit; MIMIC, Medical Information Mart for Intensive Care; PIV, pan-immune-inflammation value.


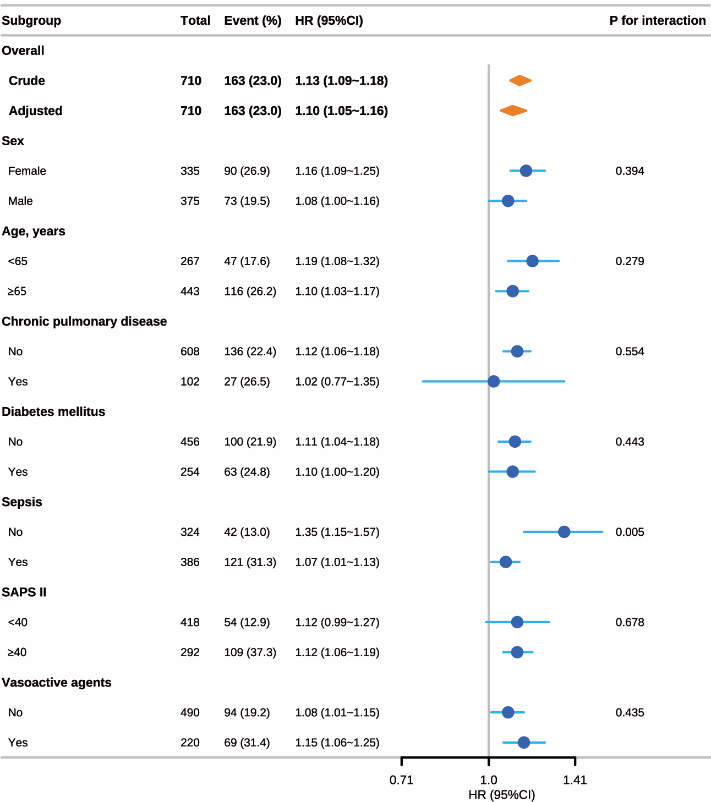


**Supplemental Figure 2** Subgroup analysis for the effect between PIV and 30-day ACM.

PIV was expressed as a continuous variable (per 1000 units). Each stratification factor was adjusted for sex, age, race, congestive heart failure, chronic pulmonary disease, diabetes mellitus, sepsis, liver disease, renal disease, malignant cancer, heart rate, SBP, DBP, MBP, respiratory rate, temperature, SpO_2_, WBC, HGB, glucose, BUN, creatinine, SAPS II, APS III, vasoactive agents and mechanical ventilation. If the stratification factor was a categorical variable, it was omitted from the subgroup analysis.

PIV, pan-immune-inflammation value; ACM, all-cause mortality; ICU, intensive care unit; AIS, acute ischemic stroke; SBP, systolic blood pressure; DBP, diastolic blood pressure; MBP, mean blood pressure; SpO_2_, peripheral capillary oxygen saturation; WBC, white blood cell count; HGB, hemoglobin; BUN, blood urea nitrogen; SAPS II, simplified acute physiology score II; APS III, acute physiology score III; HR, hazard ratio; CI, confidence interval.


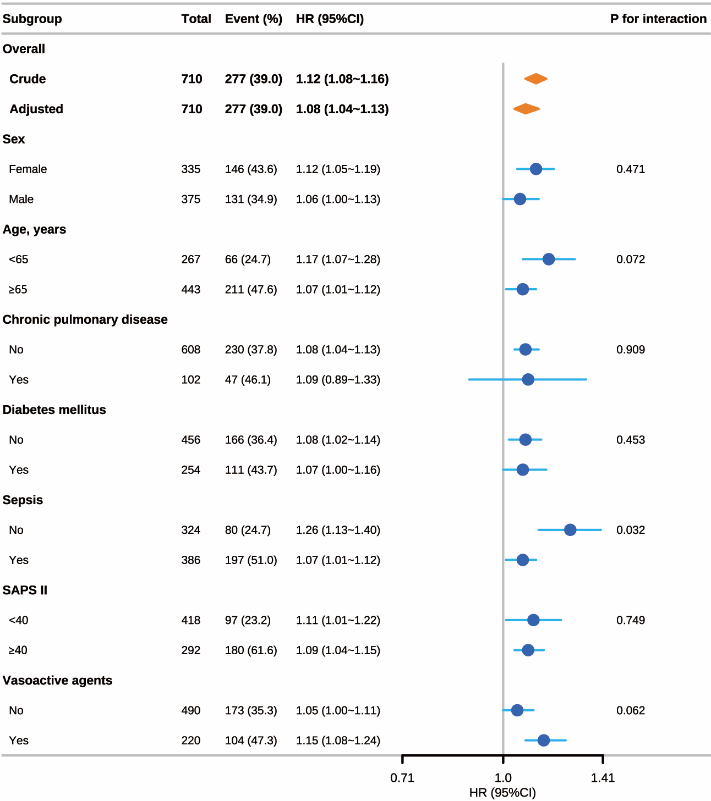


**Supplemental Figure 3** Subgroup analysis for the effect between PIV and 1-year ACM.

PIV was expressed as a continuous variable (per 1000 units). Each stratification factor was adjusted for sex, age, race, congestive heart failure, chronic pulmonary disease, diabetes mellitus, sepsis, liver disease, renal disease, malignant cancer, heart rate, SBP, DBP, MBP, respiratory rate, temperature, SpO_2_, WBC, HGB, glucose, BUN, creatinine, SAPS II, APS III, vasoactive agents and mechanical ventilation. If the stratification factor was a categorical variable, it was omitted from the subgroup analysis.

PIV, pan-immune-inflammation value; ACM, all-cause mortality; ICU, intensive care unit; AIS, acute ischemic stroke; SBP, systolic blood pressure; DBP, diastolic blood pressure; MBP, mean blood pressure; SpO_2_, peripheral capillary oxygen saturation; WBC, white blood cell count; HGB, hemoglobin; BUN, blood urea nitrogen; SAPS II, simplified acute physiology score II; APS III, acute physiology score III; HR, hazard ratio; CI, confidence interval.

**
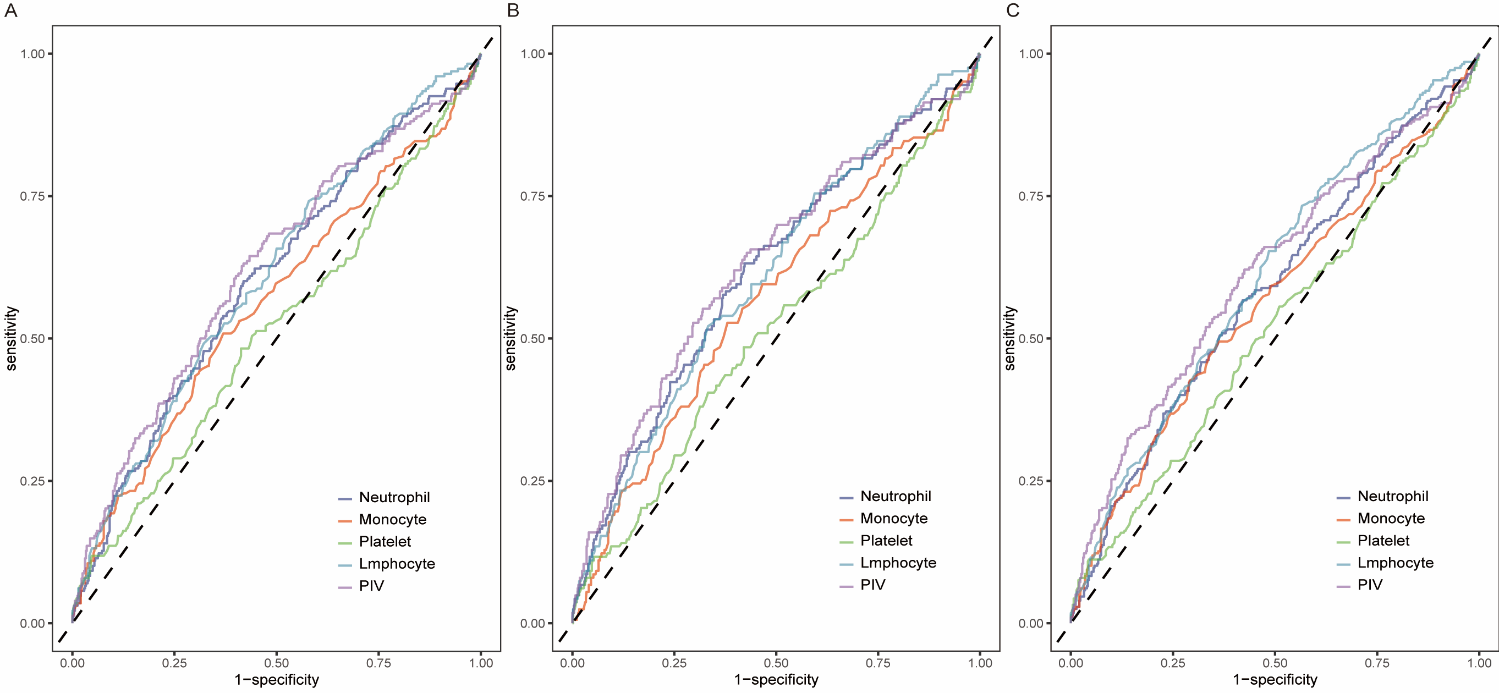
Supplemental** **Figure 4** Time-dependent receiver operating characteristic curves of PIV and its components for 90-day **(A)**, 30-day **(B)**, and 1-year **(C)** ACM in critically ill AIS patients. PIV, pan-immune-inflammation value; ACM, all-cause mortality; AIS, acute ischemic stroke.


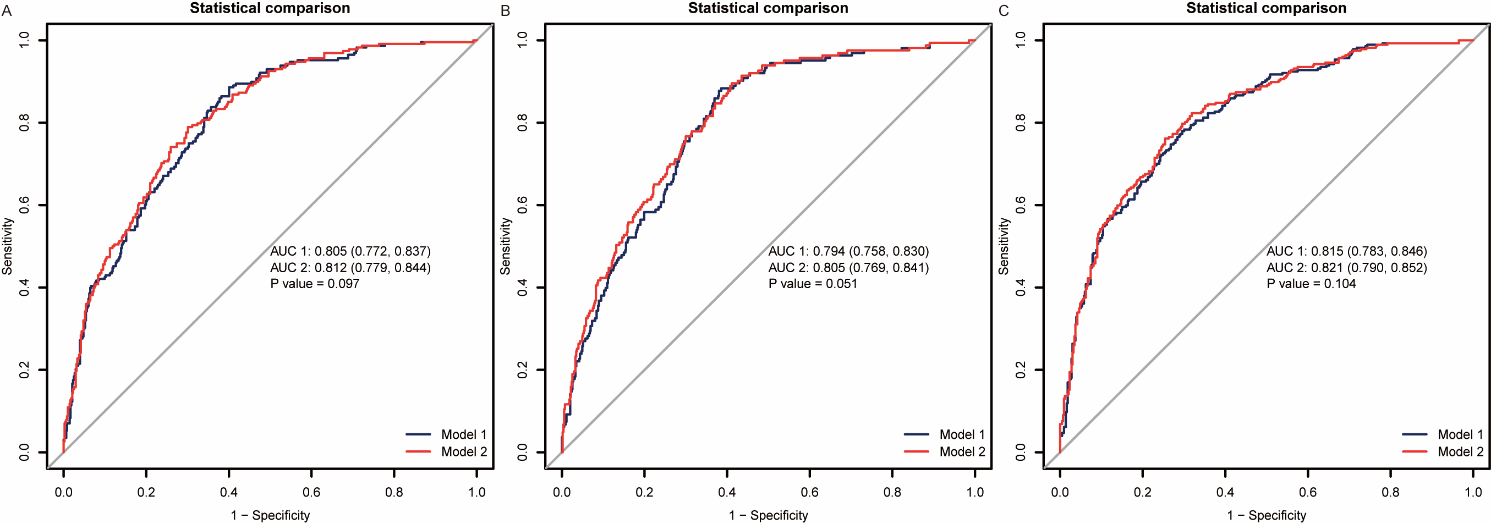


**Supplemental Figure 5** Time-dependent receiver operating characteristic curves of the models with and without PIV for 90-day **(A)**, 30-day **(B)**, and 1-year **(C)** ACM in critically ill AIS patients. Model 1 (Baseline risk model) includes age, sex, race, congestive heart failure, diabetes mellitus, chronic pulmonary disease, sepsis, liver disease, renal disease, malignant cancer, heart rate, SBP, DBP, MBP, temperature, respiratory rate, SpO_2_, WBC, HGB, glucose, creatinine, BUN, SAPS II, APS III, vasoactive agents, mechanical ventilation. Model 2 includes baseline risk model plus PIV. PIV, pan-immune-inflammation value; ACM, all-cause mortality; AIS, acute ischemic stroke; SBP, systolic blood pressure; DBP, diastolic blood pressure; MBP, mean blood pressure; SpO_2_, peripheral capillary oxygen saturation; WBC, white blood cell count; HGB, hemoglobin; BUN, blood urea nitrogen; SAPS II, simplified acute physiology score II; APS III, acute physiology score III.


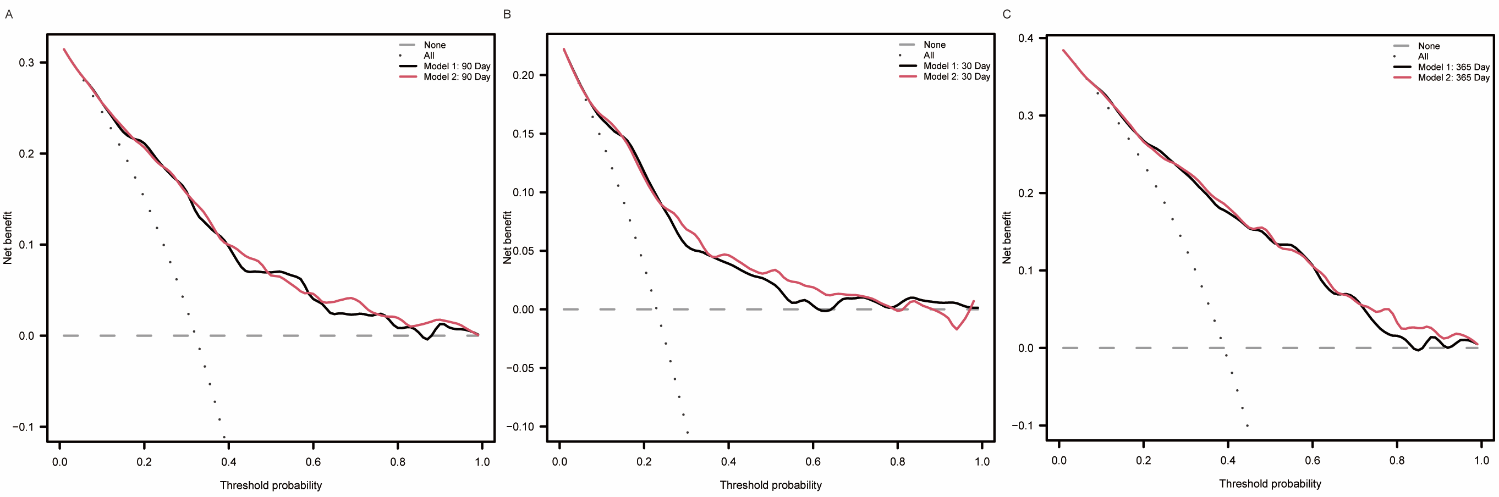


**Supplemental Figure 6** Decision curve analysis of the models with and without PIV for 90-day **(A)**, 30-day **(B)**, and 1-year **(C)** ACM in critically ill AIS patients. Model 1 (Baseline risk model) includes age, sex, race, congestive heart failure, diabetes mellitus, chronic pulmonary disease, sepsis, liver disease, renal disease, malignant cancer, heart rate, SBP, DBP, MBP, temperature, respiratory rate, SpO_2_, WBC, HGB, glucose, creatinine, BUN, SAPS II, APS III, vasoactive agents, mechanical ventilation. Model 2 includes baseline risk model plus PIV. PIV, pan-immune-inflammation value; ACM, all-cause mortality; AIS, acute ischemic stroke; SBP, systolic blood pressure; DBP, diastolic blood pressure; MBP, mean blood pressure; SpO_2_, peripheral capillary oxygen saturation; WBC, white blood cell count; HGB, hemoglobin; BUN, blood urea nitrogen; SAPS II, simplified acute physiology score II; APS III, acute physiology score III.
